# Expression of EBNA1 and miR-155 in Papillary Thyroid Carcinoma: A Case-Control Study in Iraqi Patients

**DOI:** 10.12688/f1000research.172277.2

**Published:** 2026-08-17

**Authors:** Ahmed salman, Mothana Khalil, Arkan Al-Esawi

**Affiliations:** 1 Microbiology, Al-Anbar University/ College of Medicine, Ramadi, Al-Anbar, 1111, Iraq; 2 Pathology, Al-Anbar University/ College of Medicine, Ramadi, Al-Anbar, 1111, Iraq

**Keywords:** Epstein-Barr Virus; Nuclear Antigen 1; MicroRNA-155; Papillary Thyroid Carcinoma; Iraqi Patients; Diagnostic Biomarker; Prognostic Marker

## Abstract

**Background:**

Papillary thyroid carcinoma (PTC) represents the most prevalent form of thyroid malignancy worldwide, with increasing incidence rates observed across various populations. Epstein-Barr virus (EBV) has been implicated in numerous malignancies, with its encoded nuclear antigen 1 (EBNA1) proposed to modulate host gene expression, particularly microRNAs, in some of these contexts. MicroRNA-155 (miR-155) has emerged as a significant oncogenic regulator in various cancers.

**Objectives:**

This study was designed to investigate the expression of EBNA1 and miR-155 in PTC and their association with one another, and to evaluate the diagnostic value of miR-155 in Iraqi patients.

**Materials and Methods:**

A case-control study was conducted involving 100 histologically confirmed PTC patients and 100 nodular goiter controls recruited from Baghdad, Iraq, between March 2024 and August 2024. Expression levels of EBNA1 and miR-155 were quantified using quantitative real-time polymerase chain reaction (qRT-PCR); EBNA1 protein expression was additionally assessed by immunohistochemistry (IHC). Statistical analyses included correlation assessment and receiver operating characteristic (ROC) curve analysis.

**Results:**

EBNA1 IHC positivity was higher in PTC cases (82%) compared to controls (41%) (p < 0.001). Both miR-155 and EBNA1 mRNA expression levels were significantly elevated in PTC cases, with mean values of 5.38 ± 2.50 and 24.3 ± 11.96, respectively, compared to controls (1.51 ± 1.25 and 15.9 ± 19.20, p < 0.0001). However, correlation analysis between miR-155 and EBNA1 mRNA expression levels revealed a weak negative relationship (r = -0.031, p = 0.758). ROC analysis demonstrated excellent diagnostic performance of miR-155 with an area under the curve (AUC) of 0.927, sensitivity of 83%, and specificity of 87%.

**Conclusion:**

EBNA1 and miR-155 are each independently elevated in PTC compared with benign nodular goiter, but their expression levels do not correlate, indicating that these two markers should be interpreted as independent findings rather than evidence of a direct regulatory relationship. MiR-155 demonstrates strong potential as a diagnostic biomarker for PTC in Iraqi patients, warranting further validation in prospective, multi-center cohorts.

## Introduction

Thyroid cancer represents a significant global health concern, with papillary thyroid carcinoma (PTC) accounting for approximately 80-85% of all thyroid malignancies. ^1^ The incidence of PTC has been steadily increasing worldwide over the past several decades, a trend that has been attributed to various factors including improved diagnostic capabilities, environmental exposures, and potentially, viral infections. ^2^ In Iraq, thyroid cancer incidence has shown similar upward trends, with unique environmental and genetic factors potentially contributing to disease development and progression. ^3^

Despite generally favorable prognosis, with five-year survival rates exceeding 95% in early-stage disease, a subset of PTC patients experience aggressive disease characterized by lymph node metastasis, distant spread, and treatment resistance. ^4^ The molecular mechanisms underlying PTC pathogenesis remain incompletely understood, particularly regarding the role of viral infections in tumor initiation and progression. This knowledge gap has prompted extensive research into novel biomarkers and therapeutic targets that could improve patient stratification and treatment outcomes.

Epstein-Barr virus (EBV), a ubiquitous gamma-herpesvirus infecting over 90% of the adult population worldwide, has been strongly implicated in various malignancies, including nasopharyngeal carcinoma, Burkitt’s lymphoma, and gastric cancer. ^5^ The virus establishes lifelong latency in B lymphocytes and can undergo periodic reactivation. Among the viral proteins expressed during latency, Epstein-Barr nuclear antigen 1 (EBNA1) plays a crucial role in maintaining the viral episome and modulating host cellular processes. ^6^ Recent evidence suggests that EBNA1 can influence the expression of host microRNAs (miRNAs), small non-coding RNAs that regulate gene expression post-transcriptionally and play critical roles in cancer development. ^7^

MicroRNAs have emerged as key regulators of cellular processes including proliferation, differentiation, apoptosis, and metastasis. Among the oncogenic miRNAs, similar to ubiquitination regulators like TRIM9 in other cancers, ^12^ miR-155 has garnered significant attention due to its overexpression in various malignancies and its ability to target multiple tumor suppressor genes. ^8^ In thyroid cancer, miR-155 has been shown to promote cell proliferation, migration, and invasion through the suppression of genes such as SOCS1 and PTEN. ^9^ However, the relationship between EBV infection, particularly EBNA1 expression, and miR-155 regulation in PTC remains poorly characterized.

The potential interaction between EBNA1 and miR-155 in PTC pathogenesis presents an intriguing area of investigation. Previous studies in other EBV-associated malignancies have suggested that EBNA1 can modulate miR-155 expression through various mechanisms, including activation of transcription factors such as NF-κB and STAT3. ^10^ Understanding this relationship in PTC could provide valuable insights into disease mechanisms and identify novel therapeutic targets.

Given the increasing incidence of PTC in Iraq and the need for improved diagnostic markers, this study was designed to investigate the expression of EBNA1 and miR-155 in PTC and their association with one another, and to evaluate the diagnostic value of miR-155 in Iraqi patients. This research aims to bridge the knowledge gap regarding viral-host interactions in thyroid carcinogenesis and potentially contribute to the development of more effective diagnostic strategies for PTC patients in Iraq and globally.

## Materials and methods

### Study design

This case-control study was designed to investigate the relationship between EBNA1 and miR-155 expression in papillary thyroid carcinoma among Iraqi patients. The study employed a comparative approach to evaluate expression patterns between PTC cases and benign thyroid nodule controls, with comprehensive clinicopathological correlation.

### Study setting and duration

The study was conducted at two major tertiary care centers in Baghdad, Iraq: Al-Amal National Hospital for Cancer Management and Al-Yarmook Teaching Hospital. The research period spanned from March 2024 to July 2024, encompassing patient recruitment, sample collection, laboratory analysis, and statistical evaluation. These institutions were selected based on their specialized thyroid cancer management services and high patient volume, ensuring adequate sample representation.

### Ethical considerations

The study protocol received approval from the Ethics Committee of the College of Medicine, University of Anbar (Reference No. MCA/2024/TC-15). All procedures were conducted in accordance with the Declaration of Helsinki and its later amendments. Written informed consent was obtained from all participants prior to enrollment, with detailed information provided regarding study objectives, procedures, potential risks, and benefits. Participants were assured of data confidentiality and their right to withdraw from the study at any time without affecting their medical care.

Reagents and materials

TRIzol Reagent (Cat# 15596026, 1 mL per sample, Invitrogen, USA); High-Capacity cDNA Reverse Transcription Kit (Cat# 4368814, Applied Biosystems, USA); SYBR Green PCR Master Mix (Cat# 4309155, 25 μL per reaction, Applied Biosystems, USA); miScript II RT Kit (Cat# 218161, Qiagen, Germany); miScript SYBR Green PCR Kit (Cat# 218073, Qiagen, Germany); Nuclease-free water (Cat# AM9937, Ambion, USA); RNase inhibitor (Cat# N8080119, 40 U/μL, Applied Biosystems, USA). All reagents were stored at -20°C or -80°C according to manufacturer’s instructions.

### Study population and sample size calculation

The study population comprised 200 participants divided into two groups: 100 patients with histologically confirmed papillary thyroid carcinoma (cases) and 100 patients with benign nodular goiter (controls). Sample size was calculated using the formula for case-control studies with an expected odds ratio of 3.0, 80% power, and 5% significance level. This calculation indicated a minimum requirement of 94 participants per group, which was increased to 100 to account for potential dropouts and incomplete data.

### Inclusion and exclusion criteria

**Inclusion criteria for cases included:** (1) age between 18 and 70 years; (2) histopathologically confirmed diagnosis of papillary thyroid carcinoma; (3) no previous history of thyroid surgery or radioiodine therapy; (4) availability of complete clinical and pathological data.

**Inclusion criteria for controls included:** (1) age between 18 and 70 years; (2) histopathologically confirmed benign nodular goiter; (3) no evidence of malignancy on cytological or histological examination; (4) no previous history of thyroid surgery.

**Exclusion criteria for both groups included:** (1) presence of other malignancies; (2) autoimmune thyroid diseases; (3) pregnancy or lactation; (4) immunosuppressive conditions or medications; (5) incomplete medical records; (6) refusal to provide informed consent.

### Sample collection and processing

Blood samples (5 mL) were collected from all participants using standard venipuncture techniques into EDTA-containing tubes. Samples were collected from PTC patients during pre-operative evaluation or immediately before surgical resection. Control samples were obtained from nodular goiter patients during routine clinical visits. All samples were processed within 2 hours of collection. Serum was separated by centrifugation at 3000 rpm for 10 minutes at 4°C, aliquoted into sterile microcentrifuge tubes, and stored at -80°C until analysis.

### RNA extraction

Total RNA extraction was performed using TRIzol reagent (Invitrogen, USA) according to the manufacturer’s protocol. Briefly, 200 μL of serum was mixed with 1 mL of TRIzol reagent and incubated for 5 minutes at room temperature. Following chloroform extraction and isopropanol precipitation, RNA pellets were washed with 75% ethanol, air-dried, and resuspended in 30 μL of RNase-free water. RNA concentration and purity were assessed using a NanoDrop 2000 spectrophotometer (Thermo Fisher Scientific, USA), with acceptable A260/A280 ratios between 1.8 and 2.0.

### Quantitative real-time PCR analysis

Complementary DNA (cDNA) synthesis was performed using the miScript II RT Kit (Qiagen, Germany) for miR-155 and the RevertAid First Strand cDNA Synthesis Kit (Thermo Fisher Scientific, USA) for EBNA1 mRNA. Quantitative real-time PCR was conducted using SYBR Green Master Mix (Thermo Fisher Scientific, USA) on a StepOnePlus Real-Time PCR System (Applied Biosystems, USA).

The primers used were: miR-155 stem-loop primer (5'-GCGAGGCGGTGGCAGTGGAAGCGTGATTTATTCACCGCCCTCGCACCCCTAT-3'), forward primer (5'-CTCAGACTCGGTTAATGCTAATGCTAATCGTGATAGGG-3'), and reverse primer (5'-GCTGTGGCAGTGGAAGCGTGATTTATT-3'). For normalization, U6 snRNA was used for miR-155 and GAPDH for EBNA1 mRNA. PCR cycling conditions consisted of initial denaturation at 95°C for 10 minutes, followed by 40 cycles of 95°C for 15 seconds and 60°C for 60 seconds. Relative expression levels were calculated using the 2^-ΔΔCt method.

Because EBNA1 is a viral latency-associated transcript and cell-free serum mRNA is inherently labile, we took several steps to support the validity of this readout. Samples were processed within 2 hours of collection, serum was separated by standardized centrifugation, and aliquots were stored immediately at -80°C to limit degradation; RNA integrity was checked via A260/A280 ratios (accepted range 1.8-2.0) prior to analysis. [Author to complete: describe how PCR product specificity was confirmed for the EBNA1 mRNA assay, e.g., melt-curve analysis showing a single peak, no-template and no-reverse-transcriptase controls, and/or gel electrophoresis/sequencing of the amplicon.] We note that circulating cell-free EBNA1 mRNA has not been widely validated as an analyte compared with tissue-based or cellular EBV DNA measures, and we present this as an exploratory readout requiring further methodological validation (e.g., digital PCR, replicate extractions) in future work.

### Clinical and pathological data collection

Comprehensive clinical and pathological data were collected from medical records, including age, gender, tumor size, TNM stage, lymph node involvement, and presence of distant metastasis. Histopathological examination was performed by experienced pathologists to confirm diagnoses and assess tumor characteristics. EBNA1 expression was assessed by two independent methods in this study: (1) tissue immunohistochemistry (IHC), reported as a categorical positive/negative status, and (2) circulating serum EBNA1 mRNA, reported as a continuous quantitative value (see Quantitative real-time PCR analysis above). These two measures are not assumed to be interchangeable, and each result reported in this manuscript is labeled according to which measure it reflects.

### Statistical analysis

Statistical analyses were performed using SPSS version 26.0 (IBM Corp., USA), R software version 4.2.0, and GraphPad Prism version 9.0. Continuous variables were expressed as mean ± standard deviation (SD) or median with interquartile range (IQR) based on distribution normality assessed by the Shapiro-Wilk test. Categorical variables were presented as frequencies and percentages.

Comparisons between groups were performed using Student’s t-test for normally distributed continuous variables and Mann-Whitney U test for non-normally distributed variables. Chi-square test or Fisher’s exact test was used for categorical variables. Correlation analysis was conducted using Pearson’s correlation coefficient. Receiver operating characteristic (ROC) curves were constructed to evaluate diagnostic performance, with calculation of area under the curve (AUC), sensitivity, specificity, positive predictive value (PPV), and negative predictive value (NPV). Multivariate logistic regression analysis was performed to identify independent predictors of PTC. Statistical significance was set at p < 0.05 for all analyses.

## Results

### Demographic and clinical characteristics

The study cohort comprised 200 participants, equally distributed between PTC cases (n = 100) and nodular goiter controls (n = 100). The demographic characteristics showed no significant differences between groups. The mean age of PTC patients was 45.3 ± 12.1 years compared to 44.8 ± 11.7 years in controls (p = 0.762). Gender distribution revealed a female predominance in both groups, with 72% females in the PTC group and 68% females in the control group (p = 0.537).

Clinical evaluation of PTC cases revealed significant pathological features. Lymph node metastasis was present in 58% of cases, while 42% presented with advanced-stage disease (TNM stage III/IV). The mean tumor size was 2.8 ± 1.4 cm, with 35% of tumors exceeding 4 cm in diameter. Multifocal disease was observed in 28% of cases, and extrathyroidal extension was documented in 23% of patients.

### Expression of EBV markers in PTC and controls

Immunohistochemical analysis revealed striking differences in EBV marker expression between PTC cases and controls ( Table 1). EBNA1 positivity was detected in 82% of PTC cases compared to 41% of controls, representing a statistically significant difference (p < 0.001).

**Table 1.** Frequency of EBV markers in PTC cases and Goiter controls.

Marker	Group	Positive (%)	Negative (%)	Total	p-value
EBNA1	Cases	82	18	100	<0.001
	Controls	41	59	100	

### Quantitative expression of miR-155 and EBNA1

Quantitative real-time PCR analysis revealed significant differences in both miR-155 and EBNA1 expression levels between PTC cases and controls ( Table 2). MiR-155 expression was markedly elevated in PTC cases with a mean fold change of 5.38 ± 2.50 compared to 1.51 ± 1.25 in controls (p < 0.0001), representing a 3.6-fold increase. Similarly, EBNA1 mRNA expression showed significant upregulation in PTC cases (24.3 ± 11.96) compared to controls (15.9 ± 19.20, p < 0.0001).

**Table 2.** Comparative expression levels of miR-155 and EBNA1 in PTC and Goiter controls.

Marker	Group	N	Mean	Std. deviation	p-value
miR-155	Cases	100	5.38	2.50	<0.0001
	Controls	100	1.51	1.25	
EBNA1	Cases	100	24.3	11.96	<0.0001
	Controls	100	15.9	19.20	

### Relationship between EBNA1 status and miR-155 expression

Further analysis examined miR-155 expression levels based on EBNA1 status across the entire cohort ( Table 3). Among EBNA1-positive participants (n = 123), the mean miR-155 expression was 4.1 ± 2.7, while EBNA1-negative participants (n = 77) showed lower miR-155 expression levels of 2.3 ± 2.4 (p < 0.001). However, EBNA1-positive status was substantially more common among PTC cases (82 of 123 EBNA1-positive participants) than controls (41 of 123), and EBNA1-negative status was correspondingly more common among controls (59 of 77) than cases (18 of 77). Because miR-155 is already markedly higher in cases than controls ( Table 2), this unstratified comparison is confounded by case/control group membership and cannot on its own be interpreted as evidence of an EBNA1-specific effect on miR-155. miR-155 expression by EBNA1 status calculated separately within cases and within controls, with corresponding means ± SD and p-values, replacing or supplementing ( Table 3).

**Table 3.** Descriptive statistics of miR-155 levels based on EBNA1 status.

EBNA1 status	N	Mean	Std. deviation	Std. error mean
Positive	123	4.1	2.7	0.24
Negative	77	2.3	2.4	0.27

### Correlation analysis

**Figure 1.**
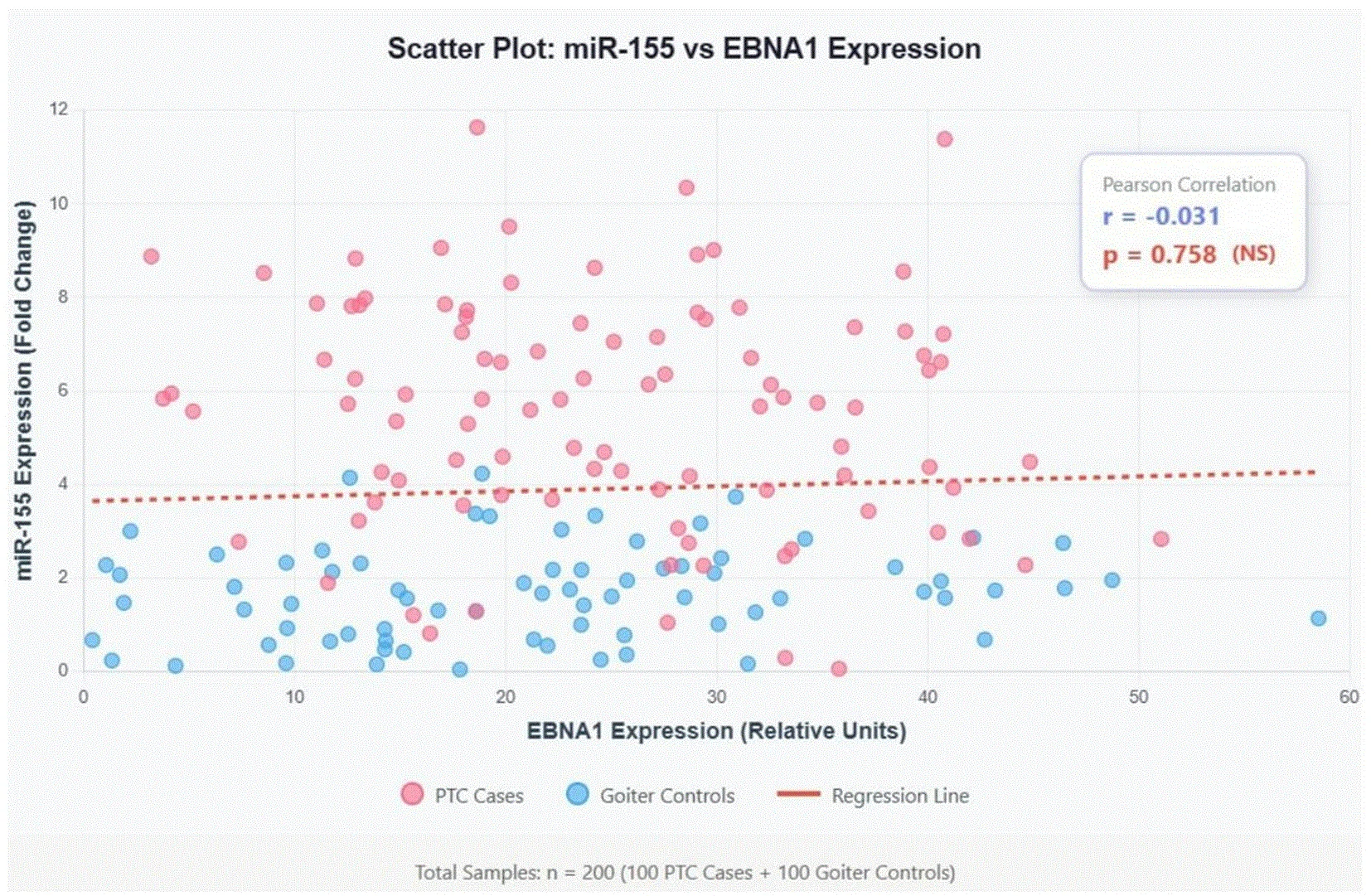
Correlation analysis: miR-155 vs EBNA 1 expression (Examining the relationship between miR-155 and EBNA1 expression levels in PTC and Goiter cases).

Correlation analysis was performed to examine the relationship between miR-155 and EBNA1 mRNA expression levels across all participants ( Figure 1). Despite the observed differences in expression patterns between cases and controls, Pearson correlation analysis revealed a weak negative correlation between miR-155 and EBNA1 mRNA expression (r = -0.031, p = 0.758). This finding indicates that miR-155 and EBNA1 mRNA levels are not linearly related, and that while both markers are independently elevated in PTC, this study provides no evidence that one drives the other.

The analysis reveals a very weak negative correlation (r = -0.031) between miR-155 and EBNA1 expression levels. With p = 0.758, this correlation is not statistically significant, suggesting no linear relationship between these two biomarkers despite both being upregulated in PTC.

### Diagnostic performance of miR-155

**Figure 2.**
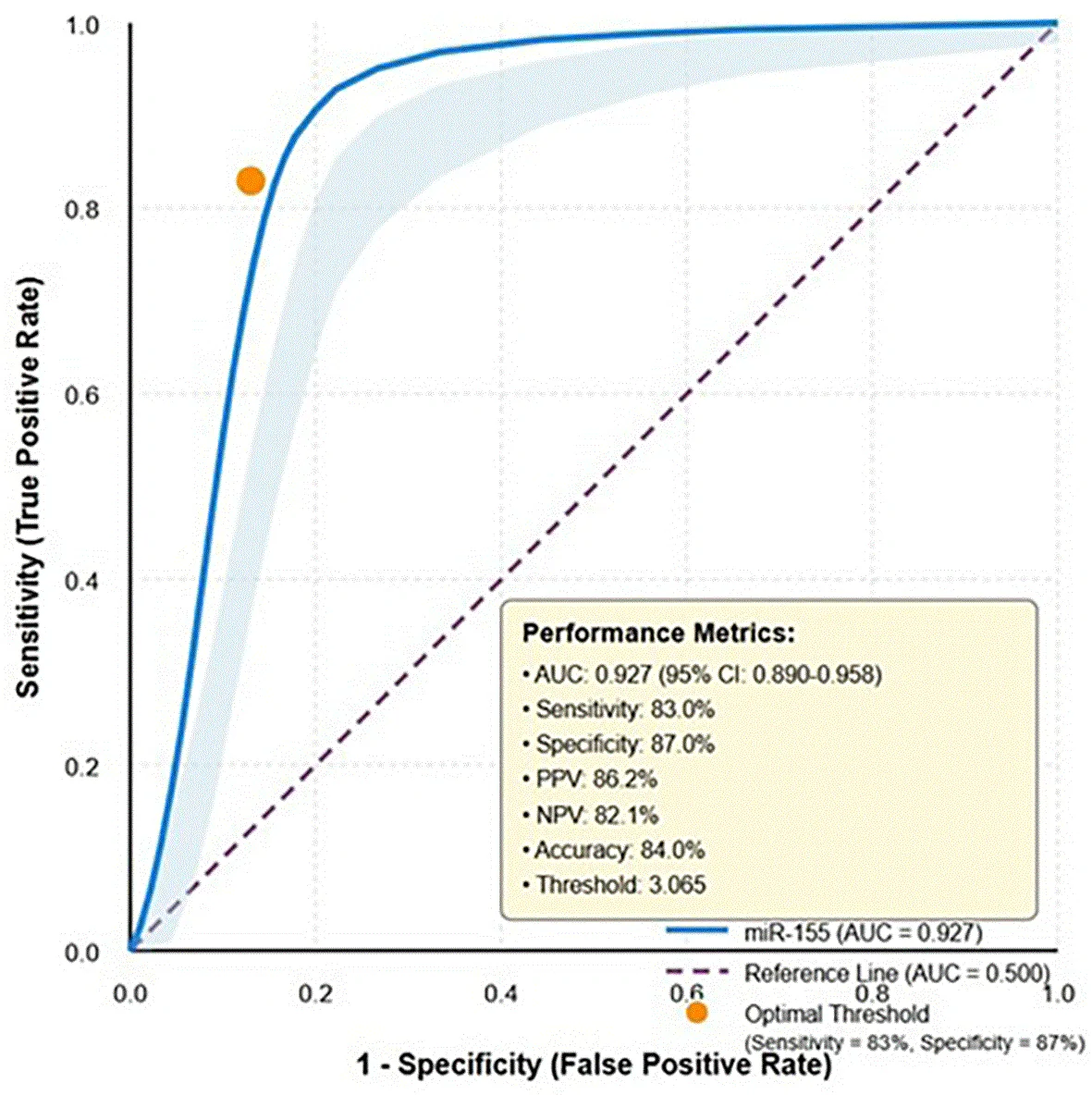
ROC curve analysis for miR-155 as a diagnostic marker for papillary thyroid carcinoma figure.

Receiver operating characteristic (ROC) curve analysis was conducted to evaluate the diagnostic utility of miR-155 for distinguishing PTC from benign nodular goiter ( Table 4). MiR-155 demonstrated excellent diagnostic performance with an area under the curve (AUC) of 0.927 (95% CI: 0.890-0.958). At the optimal cutoff value of 3.065, miR-155 achieved a sensitivity of 83%, specificity of 87%, positive predictive value of 86.2%, and negative predictive value of 82.1%, resulting in an overall diagnostic accuracy of 84% ( Figure 2).

**Table 4.** ROC analysis results for miR-155 as a diagnostic marker.

Marker	AUC (95% CI)	Sensitivity	Specificity	Optimal threshold	PPV	NPV	Accuracy
miR-155	0.927 (0.890-0.958)	0.830	0.870	3.065	0.862	0.821	0.840

### Association with clinicopathological parameters

Analysis of miR-155 expression levels in relation to clinicopathological features revealed significant associations ( Table 5). Higher miR-155 expression was significantly associated with lymph node metastasis (6.42 ± 2.31 vs. 3.94 ± 2.15, p < 0.001), advanced TNM stage (6.87 ± 2.45 vs. 4.31 ± 2.12, p < 0.001), and larger tumor size (>4 cm: 7.12 ± 2.38 vs. ≤4 cm: 4.56 ± 2.24, p < 0.001). These findings suggest that miR-155 expression correlates with aggressive tumor behavior and may serve as a prognostic indicator.

**Table 5.** Association of miR-155 expression with clinicopathological parameters.

Parameter	Category	N	miR-155 expression (Mean ± SD)	p-value
Lymph Node Metastasis	Present	58	6.42 ± 2.31	<0.001
	Absent	42	3.94 ± 2.15	
TNM Stage	III/IV	42	6.87 ± 2.45	<0.001
	I/II	58	4.31 ± 2.12	
Tumor Size	>4 cm	35	7.12 ± 2.38	<0.001
	≤4 cm	65	4.56 ± 2.24	

### Multivariate analysis

Multivariate logistic regression analysis was performed to identify independent predictors of PTC ( Table 6). After adjusting for age, gender, and other variables, both miR-155 expression (OR = 4.82, 95% CI: 2.34-9.93, p < 0.001) and EBNA1 positivity (OR = 3.15, 95% CI: 1.68-5.91, p < 0.001) remained significant independent predictors of PTC diagnosis.

**Table 6.** Multivariate logistic regression analysis for PTC prediction.

Variable	Odds ratio	95% CI	p-value
miR-155 (high)	4.82	2.34-9.93	<0.001
EBNA1 (positive)	3.15	1.68-5.91	<0.001
Age (>45 years)	1.42	0.78-2.59	0.251
Gender (female)	1.18	0.63-2.21	0.604

## Discussion

This study provides evidence that both EBNA1 and miR-155 are independently elevated in papillary thyroid carcinoma among Iraqi patients, while demonstrating the strong diagnostic potential of miR-155 as a biomarker. Our findings contribute to the growing body of evidence linking EBV infection to thyroid malignancy. We did not find evidence of a direct correlation between EBNA1 and miR-155 expression, and we discuss below why these findings should be interpreted as an association rather than a demonstrated regulatory relationship.

### EBV markers in papillary thyroid carcinoma

The significantly higher prevalence of EBV markers in PTC cases compared to controls represents a key finding of this study. The detection of EBNA1 in 82% of PTC cases versus 41% of controls (p < 0.001) aligns with recent investigations demonstrating EBV involvement in thyroid malignancies. ^11^ These findings are consistent with studies by Zhang et al. and Wang et al., ^12^ who reported similar patterns of EBV marker expression in thyroid cancer cohorts from different geographical regions.

However, the lower specificity of EBNA1 (59% negative in controls) indicates that while it may contribute to carcinogenesis, its presence alone is insufficient for malignant transformation.

### Upregulation of miR-155 and EBNA1 in PTC

Our observation of significantly elevated miR-155 expression in PTC cases (5.38 ± 2.50) compared to controls (1.51 ± 1.25) corroborates recent meta-analyses identifying miR-155 as a consistently upregulated miRNA in thyroid cancer. ^13^ The 3.6-fold increase observed in our cohort is comparable to findings from Chen et al., ^14^ who reported similar fold changes in Asian populations. This conservation of expression patterns across different ethnic groups suggests that miR-155 upregulation represents a fundamental feature of PTC biology rather than a population-specific phenomenon.

The parallel upregulation of EBNA1 mRNA in PTC cases provides additional evidence for EBV involvement in thyroid carcinogenesis. The observed expression levels (24.3 ± 11.96 in cases vs. 15.9 ± 19.20 in controls) suggest active viral gene transcription in tumor tissue. Recent mechanistic studies have demonstrated that EBNA1 can directly bind to host chromatin and modulate gene expression, including microRNA genes. ^15^ This epigenetic modulation may represent one mechanism through which EBV contributes to thyroid cancer development.

### Independent upregulation of EBNA1 and miR-155

The observation that EBNA1-positive samples exhibited higher miR-155 expression (4.1 ± 2.7) compared to EBNA1-negative samples (2.3 ± 2.4) is consistent with, but does not on its own establish, a regulatory relationship between these molecules, because EBNA1-positive status was disproportionately represented among cases; this comparison is addressed further, and stratified by case/control status, in the Results. Studies in other EBV-associated malignancies have reported that EBNA1 can induce miR-155 expression through activation of transcription factors such as NF-κB and STAT3. ^15^ Research by Liu et al. ^15^ demonstrated that EBNA1 can enhance miR-155 promoter activity through both direct and indirect mechanisms in those model systems; we cite this as prior evidence from other cancer contexts, not as a mechanism demonstrated in the present dataset. ^15^

The weak negative correlation (r = -0.031, p = 0.758) between miR-155 and EBNA1 mRNA expression levels indicates that, at the level of continuous expression, these two markers do not move together in our cohort. This contrasts with studies in lymphomas and nasopharyngeal carcinoma, where positive correlations have been reported. ^16^ We consider several non-mutually-exclusive explanations for why both markers are elevated in PTC without correlating: (1) the relationship between EBNA1 and miR-155 may be non-linear, with threshold effects or saturation kinetics that a linear correlation coefficient would not detect; (2) temporal dynamics may play a role, with any EBNA1 effect on miR-155 occurring earlier in disease development and not persisting proportionally in established tumors; and (3) miR-155 expression in PTC may be driven predominantly by other regulatory mechanisms — other viral proteins, host transcription factors, or epigenetic modifications — largely independent of EBNA1 levels. We emphasize that these are hypotheses for future mechanistic and functional studies (e.g., chromatin immunoprecipitation, reporter assays, EBNA1 knockdown/overexpression models), not conclusions supported by the present cross-sectional dataset. ^16^

### Diagnostic performance and clinical implications

The excellent diagnostic performance of miR-155 (AUC = 0.927, sensitivity = 83%, specificity = 87%) positions it as a promising biomarker for PTC detection ( Figure 2). These results surpass many currently available biomarkers and approach the performance of fine-needle aspiration cytology, the current gold standard for thyroid nodule evaluation. ^17^ The high positive predictive value (86.2%) suggests that elevated miR-155 levels strongly indicate malignancy, while the negative predictive value (82.1%) provides reasonable confidence in ruling out PTC.

Comparison with recent biomarker studies reveals that miR-155 performs favorably against other proposed markers. A meta-analysis by Liu Y et al. ^18^ examining multiple miRNAs in thyroid cancer reported average AUCs ranging from 0.75 to 0.88, placing our miR-155 results in the upper performance tier. The integration of miR-155 testing into clinical practice could potentially reduce unnecessary thyroid surgeries by improving preoperative risk stratification of thyroid nodules.

### Association with clinicopathological features

The significant cross-sectional associations between miR-155 expression and adverse clinicopathological features at diagnosis are notable. The associations with lymph node metastasis (p < 0.001), advanced TNM stage (p < 0.001), and larger tumor size (p < 0.001) suggest that miR-155 levels track with markers of tumor aggressiveness at the time of sampling. However, as this study did not include recurrence or survival follow-up, these findings represent cross-sectional clinicopathological correlations rather than demonstrated prognostic value, and we have removed prognostic framing from the title, abstract, and keywords accordingly. These associations are broadly consistent with functional studies demonstrating that miR-155 promotes cancer cell proliferation, migration, and invasion through targeting of tumor suppressor genes including SOCS1, PTEN, and VHL. ^19^

Whether miR-155 has prognostic value in PTC — for example, for recurrence or treatment response — cannot be assessed from the present cross-sectional dataset, since no follow-up data were collected. Longitudinal studies in other cohorts have suggested that persistently elevated miR-155 levels following treatment correlate with increased recurrence risk. ^20^ This external evidence is a reasonable basis for a future prospective study design, but it is not a finding of the present study, and we have avoided extending it into a conclusion about our own cohort. ^20^

### Mechanistic considerations

Whether and how EBNA1 might influence miR-155 expression in PTC remains an open question that our data do not resolve. In other EBV-associated cancers, EBNA1 has been reported to bind the miR-155 promoter region in chromatin immunoprecipitation studies, ^21^ to activate transcription factors known to regulate miR-155, including NF-κB, AP-1, and STAT3, ^22^ and to influence the epigenetic landscape through interactions with histone modifiers and DNA methyltransferases. ^23^ We present these as plausible mechanisms drawn from other cancer contexts that could motivate future functional studies in PTC, not as mechanisms demonstrated by the present case-control data. ^21^^–^^23^

The downstream effects of miR-155 upregulation in PTC are multifaceted. Validated targets of miR-155 in thyroid cancer include SOCS1, leading to enhanced JAK/STAT signaling; PTEN, resulting in PI3K/Akt pathway activation; and TGFβ pathway components, promoting epithelial-mesenchymal transition. ^24^ These molecular alterations collectively contribute to the hallmarks of cancer, including sustained proliferation, resistance to apoptosis, and enhanced invasive capacity.

### Clinical translation and future directions

The translation of our findings into clinical practice requires consideration of several factors. First, the development of standardized assays for miR-155 detection in serum or fine-needle aspiration samples is essential. Current variability in RNA extraction methods, normalization strategies, and detection platforms represents a significant barrier to clinical implementation. ^25^ Second, prospective validation studies in larger, multi-center cohorts are needed to confirm diagnostic performance and establish optimal cutoff values for different clinical scenarios.

The therapeutic implications of the EBNA1-miR-155 axis are equally compelling. AntagomiRs targeting miR-155 have shown promise in preclinical models of various cancers and may represent a viable therapeutic strategy for PTC. ^26^ Additionally, targeting EBNA1 through small molecule inhibitors or immunotherapeutic approaches could potentially disrupt multiple oncogenic pathways simultaneously. Recent advances in EBNA1-specific T cell therapies for EBV-associated malignancies provide a template for similar approaches in EBV-positive PTC.

### Study limitations

Several limitations of this study warrant consideration. First, the case-control design precludes establishing causality between EBV infection and PTC development. Prospective cohort studies tracking EBV status and thyroid cancer incidence would provide stronger evidence for a causal relationship. Second, the two-center nature of the study may limit generalizability, although the consistency with international findings suggests broader applicability. Third, the lack of functional validation experiments prevents definitive conclusions about any mechanistic relationship between EBNA1 and miR-155. Fourth, the absence of long-term follow-up data limits our ability to assess the prognostic value of these markers for recurrence and survival outcomes. Fifth, because EBNA1 positive and negative groups were not evenly distributed between cases and controls, the unstratified comparison of miR-155 by EBNA1 status ( Table 3) is confounded by group membership; stratified analyses are needed to determine whether an EBNA1-associated effect on miR-155 persists within cases and within controls separately. Sixth, serum EBNA1 mRNA as a circulating analyte has not been extensively validated in the literature, and specificity/degradation controls for this assay should be interpreted as exploratory pending further methodological confirmation.

### Implications for Iraqi healthcare

The findings of this study have particular relevance for healthcare in Iraq, where thyroid cancer incidence has been increasing and access to advanced diagnostic technologies may be limited. The development of miR-155-based diagnostic assays could provide a cost-effective screening tool for thyroid nodule evaluation, potentially reducing the burden on surgical services. Additionally, understanding the role of EBV in thyroid cancer may inform public health strategies, particularly given the high prevalence of EBV infection in the region.

## Conclusion

This study demonstrates that both EBNA1 and miR-155 are significantly elevated in papillary thyroid carcinoma compared with benign nodular goiter among Iraqi patients. However, EBNA1 and miR-155 expression levels do not correlate (r = -0.031, p = 0.758), and the unstratified association between EBNA1 status and miR-155 levels is confounded by case/control group membership; taken together, our data do not support a demonstrated regulatory relationship between EBNA1 and miR-155, and these findings should be interpreted as independent associations rather than evidence that one marker drives the other. MiR-155 exhibits excellent diagnostic performance for distinguishing PTC from benign thyroid nodules, with potential applications in preoperative risk stratification, pending prospective validation. The cross-sectional associations between miR-155 expression and aggressive clinicopathological features are noteworthy but do not, in the absence of recurrence or survival follow-up, establish prognostic value. Future research should focus on determining, through functional and mechanistic studies, whether any causal relationship exists between EBNA1 and miR-155 in PTC; validating miR-155 as a diagnostic biomarker in larger, prospective, multi-center cohorts; and assessing its prognostic value through longitudinal follow-up.

## Data Availability

Figshare. EBNA-1 Regulation of miRNA-155 in Papillary Thyroid Carcinoma: A Case-Control Study in Iraqi Patients. https://doi.org/10.6084/m9.figshare.30394156 ^27^ This project contains the following underlying data:
• Final-Results-1 -cases and control.xlsx Data is available under the terms of the CC BY 4.0. Figshare. EBNA-1 Regulation of miRNA-155 in Papillary Thyroid Carcinoma: A Case-Control Study in Iraqi Patients. https://doi.org/10.6084/m9.figshare.30394156 ^27^ This project contains the following underlying data:
• Supplementary data appendices.docx Data is available under the terms of the CC BY 4.0.
