# Supplementary material for: Expression of EBNA1 and miR-155 in Papillary Thyroid Carcinoma: A Case-Control Study in Iraqi Patients
Source: F1000Res. 2026 Aug 17;14:1433. Originally published 2025 Dec 23. [Version 2] doi: 10.12688/f1000research.172277.2 (PMC13560053; doi:10.12688/f1000research.172277.2)
Supplement: Peer review history

# Reviewer report: Reviewer response for version 2

**DOI:** 10.5256/f1000research.208411.r511994

**Published:** 2026-08-19

**Authors:** Arwa Mujahid Al-Shuwaikh

**Affiliations:** 1 Microbiology, College of Medicine, Al-Nahrain University, Kadhimiya, Baghdad, Iraq

Comments to Authors:

 I have re-reviewed this manuscript and am satisfied with the revisions. The authors have effectively addressed many point from my first evaluation and presented an interesting exploratory analysis; however, critical methodological details essential for experimental reproducibility are currently missing from the manuscript.

 First, in the 'Quantitative real-time PCR analysis' section, while the miR‑155 primer sequences and cycling parameters are fully provided, the forward and reverse primer sequences for the EBNA1 mRNA assay, along with its specific annealing/extension conditions (if they differ from the stated universal cycling program), are completely omitted.  Second, regarding the immunohistochemistry findings for EBNA1, the Materials and Methods lack a dedicated IHC subsection. To allow full evaluation and replication, the authors should clearly state the primary antibody used, including the clone, manufacturer, and the precise dilution/titer applied. Addressing these points will greatly enhance the transparency, validity, and reproducibility of the study.

 Best Regards

Is the work clearly and accurately presented and does it cite the current literature?

Yes

If applicable, is the statistical analysis and its interpretation appropriate?

Yes

Are all the source data underlying the results available to ensure full reproducibility?

Partly

Is the study design appropriate and is the work technically sound?

Yes

Are the conclusions drawn adequately supported by the results?

Partly

Are sufficient details of methods and analysis provided to allow replication by others?

Partly

Reviewer Expertise:

Medical Microbiology/ Virology

I confirm that I have read this submission and believe that I have an appropriate level of expertise to confirm that it is of an acceptable scientific standard.

# Reviewer report: Reviewer response for version 2

**DOI:** 10.5256/f1000research.208411.r511993

**Published:** 2026-08-19

**Authors:** Basim Mohammed Khashman

**Affiliations:** 1 University of Baghdad, Baghdad, Iraq

Approved for indexing.

Is the work clearly and accurately presented and does it cite the current literature?

Partly

If applicable, is the statistical analysis and its interpretation appropriate?

Partly

Are all the source data underlying the results available to ensure full reproducibility?

Yes

Is the study design appropriate and is the work technically sound?

Yes

Are the conclusions drawn adequately supported by the results?

Partly

Are sufficient details of methods and analysis provided to allow replication by others?

Partly

Reviewer Expertise:

Virology , Immunology

I confirm that I have read this submission and believe that I have an appropriate level of expertise to confirm that it is of an acceptable scientific standard.

# Reviewer report: Reviewer response for version 1

**DOI:** 10.5256/f1000research.189993.r504205

**Published:** 2026-08-10

**Authors:** Basim Mohammed Khashman

**Affiliations:** 1 University of Baghdad, Baghdad, Iraq

**Summary**

 Well-motivated study of an understudied population. The central findings support these; both EBNA1 and miR-155 are increased in PTC and miR-155 discriminates between cancer and goiter effectively (AUC ≈ 0.93). The paper reads as if the scientists are demonstrating that EBNA1 regulates miR-155, but the data do not support this. This is a good contribution for the work on the framing and tidying a few methods gaps. 

**Major points**

 EBNA1 and miR-155 don't correlate (r = −0.031, p = 0.758), so the paper can't say one drives the other, and "regulatory role despite no correlation" is a contradiction rather than a nuance. Retitle it as an association ("Expression of EBNA1 and miR-155 in PTC") and report what you found: both are elevated in PTC, but their levels move independently.

 EBNA1 is measured two ways, tissue IHC (positive/negative) and circulating mRNA. Table 3 uses the IHC status while the r = −0.031 uses the mRNA, and the text moves between them without flagging it. Please be explicit about which measure supports which claim.

 The EBNA1-positive group is mostly cases and the negative group mostly controls, and cases already have much higher miR-155, so the "higher miR-155 in EBNA1-positive samples" result is really just cases versus controls again. Redo it within cases and within controls separately. If the effect disappears there, the interpretation needs to change.

 There's no follow-up for recurrence or survival, so prognosis can't be assessed. Table 5 is a cross-sectional association with tumour features, not prognostic data. Reframe it accordingly and remove "prognostic" from the title, abstract, and keywords.

 The 82% versus 41% positivity is a central result, but the antibody clone, dilution, staining and scoring protocol, and positivity cut-off are all missing. Anti-EBNA1 antibodies vary widely in specificity, so without these details, and ideally a positive and negative control tissue, the figure can't be reproduced. A short IHC methods subsection would cover it.

 Measuring a viral latency transcript in serum or plasma is unusual, and cell-free mRNA is fragile, so the manuscript should explain why the readout is valid and how specificity was confirmed.

Is the work clearly and accurately presented and does it cite the current literature?

Partly

If applicable, is the statistical analysis and its interpretation appropriate?

Partly

Are all the source data underlying the results available to ensure full reproducibility?

Yes

Is the study design appropriate and is the work technically sound?

Yes

Are the conclusions drawn adequately supported by the results?

Partly

Are sufficient details of methods and analysis provided to allow replication by others?

Partly

Reviewer Expertise:

Virology , Immunology

I confirm that I have read this submission and believe that I have an appropriate level of expertise to confirm that it is of an acceptable scientific standard, however I have significant reservations, as outlined above.

# Reviewer report: Reviewer response for version 1

**DOI:** 10.5256/f1000research.189993.r445579

**Published:** 2026-01-24

**Authors:** Arwa Mujahid Al-Shuwaikh

**Affiliations:** 1 Microbiology, College of Medicine, Al-Nahrain University, Kadhimiya, Baghdad, Iraq

Report

**Title: **"EBNA-1 Regulation of miRNA-155 in Papillary Thyroid Carcinoma: A Case-Control Study in Iraqi Patients

 This manuscript explores the potential role of EBV and miRNA dysregulation in PTC within an understudied population. The study has a solid foundation and addresses an important knowledge gap. With **minor revisions** to enhance clarity, precision, and methodological reporting, it has the potential to be a valuable contribution to the literature. The following points for correction are offered to help the authors strengthen the manuscript's impact, rigor, and reproducibility.

**Points for Correction and Strengthening:**

**Title:** A title such as "Epstein-Barr Virus Nuclear Antigen 1 (EBNA-1) Regulation of miRNA-155..." would instantly clarify the viral context for a broad readership.

**Objective:** The stated objective includes evaluating the 'prognostic value' of miR-155. However, as the study design is a case-control and does not include longitudinal follow-up data on patient outcomes (e.g., survival, recurrence), it cannot assess prognosis. I recommend revising the objective to focus solely on "diagnostic value and clinicopathological associations" to accurately reflect the study's design and findings.

**Data Interpretation:** The correlation analysis (r = -0.031, p = 0.758) is a key finding. The result (r = -0.031, p = 0.758) is definitive. An 'r' value so close to zero indicates no linear relationship. The high p-value (> 0.05) confirms this "correlation" is statistically insignificant and almost certainly due to random chance. To avoid potential overinterpretation, the conclusion could be refined to state that both EBNA1 and miR-155 are upregulated in PTC but their expression levels are not linearly correlated in this cohort. This presents a more nuanced and data-supported discussion point about their potential roles in pathogenesis.

**Conclusion:** To state that EBNA1 "plays a regulatory role... *despite* the absence of a direct linear correlation" is illogical. A *direct regulatory role* on expression levels is fundamentally what a correlation analysis (like Pearson's r) is designed to detect. If one molecule regulates another, you would expect a significant statistical relationship (positive or negative) between their measured levels in the same samples. The word "despite" implies the conclusion is true even though the data doesn't show it. This is not how scientific inference works. Conclusions must be *supported by* the data, not held *in "spite of* it".

**Methodological Section:**

**-Sample collection and processing:** A minor but important clarification is needed in the Methods. Blood collected in EDTA tubes yields plasma, whereas serum comes from clotted blood. Correcting this term in the entitle manuscript is essential for exact protocol replication.

**-In the sections "Reagent and materials" and "quantitative real time PCR analysis"**: The same kits was mentioned but for different companies; we suggest listing all materials in one paragraph and reviewing the origin of each material.

**-For immunohistochemistry results:** IHC findings for EBNA1 are intriguing. To allow full evaluation and replication, the addition of a concise IHC methods subsection detailing the antibody (clone, dilution), staining protocol, scoring system, and statistical handling of the scores would complete this valuable part of the analysis.

**Terminology:** To align with best practices in scientific reporting, replacing "gender" with "sex" when referring to the biological variable of the patient cohort would enhance terminological precision. In addition add the full name to all the abbreviation used for first time  in the manuscript.

**Discussion section**: the author should pointed to the number of table or figure of the results that discussed.

**The limitations** of  the manuscript need to be rewrite more clearly.

**Thank you for the opportunity to evaluate this work.**

Is the work clearly and accurately presented and does it cite the current literature?

Yes

If applicable, is the statistical analysis and its interpretation appropriate?

Yes

Are all the source data underlying the results available to ensure full reproducibility?

Partly

Is the study design appropriate and is the work technically sound?

Yes

Are the conclusions drawn adequately supported by the results?

Partly

Are sufficient details of methods and analysis provided to allow replication by others?

Partly

Reviewer Expertise:

Medical Microbiology/ Virology

I confirm that I have read this submission and believe that I have an appropriate level of expertise to confirm that it is of an acceptable scientific standard.
